# *NOP53* as A Candidate Modifier Locus for Familial Non-Medullary Thyroid Cancer

**DOI:** 10.3390/genes10110899

**Published:** 2019-11-07

**Authors:** Aida Orois, Sudheer K. Gara, Mireia Mora, Irene Halperin, Sandra Martínez, Rocio Alfayate, Electron Kebebew, Josep Oriola

**Affiliations:** 1 Department of Endocrinology and Nutrition, ICMDM, Hospital Clinic, 08036 Barcelona, Spain; mporta@clinic.cat (M.M.); halperin@clinic.cat (I.H.); 2 Thoracic Surgery Branch, National Cancer Institute, National Institutes of Health, Bethesda, MD 20892, USA; sudheer.gara@nih.gov; 3 Faculty of Medicine, University of Barcelona, 08007 Barcelona, Spain; joriola@clinic.cat; 4 Department of Endocrinology and Nutrition, Hospital de Elda, 03600 Elda, Alicante, Spain; smartinez080l@cv.gva.es; 5 Department of Endocrinology and Nutrition, Hospital General Universitario de Alicante, 03010 Alicante, Spain; alfayate_roc@gva.es; 6 Department of Surgery and Stanford Cancer Institute, Stanford University, Stanford, CA 9430, USA; kebebew@stanford.edu; 7 Department of Biochemistry and Molecular Genetics, CDB, Hospital Clínic, 08036 Barcelona, Spain

**Keywords:** thyroid cancer, genetic abnormalities, molecular testing, *NOP53*, oncogenic mutations

## Abstract

Nonsyndromic familial non-medullary thyroid cancer (FNMTC) represents 3–9% of thyroid cancers, but the susceptibility gene(s) remain unknown. We designed this multicenter study to analyze families with nonsyndromic FNMTC and identify candidate susceptibility genes. We performed exome sequencing of DNA from four affected individuals from one kindred, with five cases of nonsyndromic FNMTC. Single Nucleotide Variants, and insertions and deletions that segregated with all the affected members, were analyzed by Sanger sequencing in 44 additional families with FNMTC (37 with two affected members, and seven with three or more affected members), as well as in an independent control group of 100 subjects. We identified the germline variant p. Asp31His in *NOP53* gene (rs78530808, MAF 1.8%) present in all affected members in three families with nonsyndromic FNMTC, and not present in unaffected spouses. Our functional studies of *NOP53* in thyroid cancer cell lines showed an oncogenic function. Immunohistochemistry exhibited increased NOP53 protein expression in tumor samples from affected family members, compared with normal adjacent thyroid tissue. Given the relatively high frequency of the variant in the general population, these findings suggest that instead of a causative gene, *NOP53* is likely a low-penetrant gene implicated in FNMTC, possibly a modifier.

## 1. Introduction

Thyroid cancer (TC) is the most common endocrine cancer, with more than 54,000 new cases diagnosed every year in the USA. In fact, it is expected that in a few years it will become the third most common cancer among American women [1].

There are two main types of TC, defined according to their cellular origin: (a) non-medullary cancer, arising from follicular cells, which accounts for 95% of TCs (mostly papillary thyroid cancer (PTC) and follicular thyroid cancer (FTC)); and (b) medullary thyroid carcinomas (MTC), which originates from parafollicular C-cells (accounting for 5% of TCs). Familial non-medullary thyroid cancer (FNMTC) represents 3–9% of TCs [2]. FNMTC is classified as either syndromic or nonsyndromic. Susceptibility genes involved in syndromic FNMTC are known: *APC* in familial adenomatous polyposis [MIM: 175100], *PTEN* in Cowden’s disease [MIM: 158350], *PRKAR1A* in Carney complex type 1 [MIM: 160980], *WRN* in Werner’s syndrome [MIM: 277700], and *DICER1* in the DICER1 syndrome [MIM: 606241], [3]. Nonsyndromic FNMTC accounts for more than 95% of all FNMTC cases and is defined by the presence of TC in two or more first-degree relatives, in the absence of environmental causes (e.g., exposure to radiation) [4]. Most cases of FNMTC are nonsyndromic and the genetic causes are still unknown [5,6]. In addition, some studies have suggested that nonsyndromic FNMTC is more aggressive than sporadic non-medullary thyroid cancer [7]. Therefore, given the prevalence and aggressiveness of nonsyndromic FNTMC, it is crucial to identify the susceptibility genes involved.

Most nonsyndromic FNMTCs show an autosomal dominant pattern of inheritance with incomplete penetrance. Given the high prevalence of TC in the general population, Charkes [8] estimated that about 62% of families with two cases of FNMTC can be phenocopies (two sporadic cases associated by chance) and, therefore, only 38% would be truly hereditary. However, if there are three affected cases, the probability of its being hereditary rises to 96%.

Different strategies have been used to identify candidate genes responsible for FNMTC: Linkage [9,10], Genome Wide Association Studies (GWAS) [11], and next generation sequencing studies [12,13]. Several candidate regions, such as PRN (1q21), NMTC1 (2q21), FTEN (8p23), MNG1 (14q32) and TCO (19p13.2), as well as candidate genes, like *SRGAP1*, *NKX2*, *FOXE1*, *HABP2* or *MAP2K5* have been suggested, but none have been clearly validated as causative of familial thyroid cancer [14,15,16]. Overall, this suggests that many genes could be involved in FNMTC in a monogenic fashion with different penetrance levels, without ruling out the possibility of polygenic inheritance (the sum of genetic variants). 

We designed this multicentric study to analyze families with FNMTC and identify putative susceptibility genes for nonsyndromic FNMTC. 

## 2. Materials and Methods 

### 2.1. Study Subjects

We designed a multicentric study in Spain to collect blood specimens (15 mL of whole blood in potassium EDTA tubes), and clinical data from families with at least two members with non-medullary thyroid cancer, confirmed by histology, without history of other malignancies, and without clinical characteristics suggestive of syndromic FNMTC. We obtained blood samples and clinical data from 45 families with nonsyndromic FNMTC (37 with two affected members and eight families with three or more affected members) from 15 hospitals in Spain. 

This project was approved by the Ethics Committee of the Hospital Clínic of Barcelona, Spain (Reg. HCB/2016/0200), and was conducted in accordance with the Declaration of Helsinki. Patients gave written informed consent before undergoing evaluation and testing.

### 2.2. DNA Extraction, Exome Capture and Next-Generation Sequencing

Genomic DNA was extracted from peripheral blood samples (using conventional salt-precipitation protocol). The DNA library was prepared using the SureSelect exon v5-post kit (Agilent Technologies, Santa Clara, CA, USA), that enables the capture of the target sequence of exonic regions in the human genome. The libraries were sequenced using the Illumina HiSeq 2000 sequencer (Macrogene, Seoul, Korea), with 101-base pair (bp) average read length.

**Figure 1:**
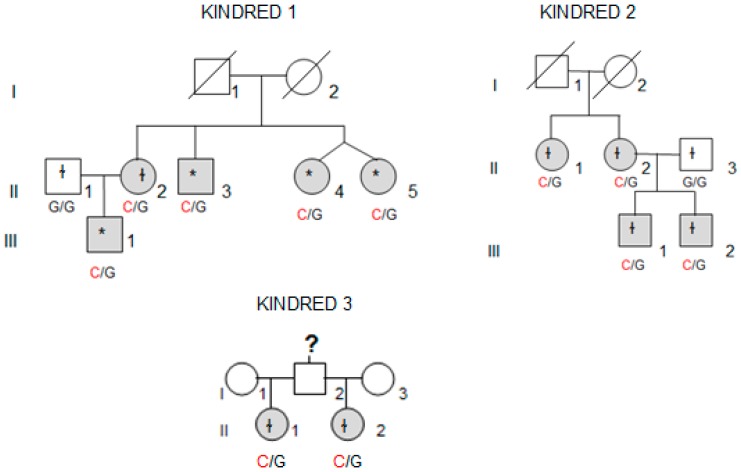
Family pedigrees for Kindreds 1, 2 and 3 and the *NOP53* genotype for each heterozygous mutation (c.91G > C, p. Asp31His). Patients affected by thyroid cancer are shown in grey. The asterisk indicates p. Asp31His variant was observed in whole-exome sequencing (WES) and validated by Sanger sequencing, whereas ɬ indicates that the variant was identified using direct Sanger sequencing.

Whole-exome sequencing (WES) was performed in four affected individuals (marked with an asterisk in Figure 1) from the kindred with the most affected members (Kindred 1, with five affected cases; II.4 and II.5 are dizygotic twins). 

### 2.3. Filtering Criteria of Whole-Exome Sequencing Data and Validation

We filtered and identified germline single nucleotide variants (SNVs) and insertions/deletions (INDELs) using HaplotypeCaller and The Genome Analysis Toolkit website [17]. The sequence data was mapped to the human reference genome (GRCh37/hg19) using Burrows–Wheeler Alignment (BWA) method. We used the following filters (Table 1): present in heterozygosis with coverage >20 in all affected members of Kindred 1, present in exonic region, nonsynonymous or frameshift deletion/insertion changes, SIFT score less than 0.05 or not available, Minor Allele Frequency (MAF) in the European Non-Finish population less than 2% in Exome Aggregation Consortium (ExAC) browser and 1000 Genome databases [18,19]; genes described as tumor suppressor genes (TSG) or proto-oncogenes, and present in all affected members in at least another kindred.

We identified five likely pathogenic variants (LPV), but only two were confirmed by Sanger sequencing. These two LPV were then studied in our 45 families (included Kindred 1, to confirm the WES results), and in our own independent control group (100 anonymous subjects from the blood donor bank). For that purpose, PCR, followed by Sanger sequencing, was performed. PCR conditions were as follows: denaturation at 95 °C for 5 min, 10 cycles (95 °C for 1 min, 65–60 °C for 1 min, 72 °C for 1 min), followed by 25 cycles (95 °C for 1 min, 55 °C for 1 min, 72 °C for 1 min) and extension at 72 °C (10 min). Sanger sequencing was performed using the following primers (Sigma–Aldrich, Saint Louis, MO, USA):

*NOP53*, exon 1. Forward TTGGTGGGAAGCGCAGCTCG
*NOP53*, exon 1. Reverse TCCAGGAACTGGTCAACCTC
*SH3BP1*, exon 7. Forward CCCATGTGGTCTGCTGTATG
*SH3BP1*, exon 7. Reverse AAGAGGCTACGAGGGAATGG

Sequences were analyzed using the CodonCode Aligner software version 7.0. (CodonCode Co., USA).

### 2.4. Cell Culture

Three cell lines were used in this study: TPC1 (papillary thyroid cancer cell line) was obtained from Dr. Nabuo Satoh (Japan). FTC133 (follicular thyroid cancer cell line) was kindly provided by Dr. Peter Goretzki (Germany). BCPAP (female derived, papillary thyroid cancer (PTC) cell line with a *BRAF* V600E and *TP53* D259Y mutations) was purchased from American Type Culture Collection (ATCC; Rockville, MD, USA). All cell lines were authenticated by short-tandem repeat profiling. All experiments were done using cell lines between passages P8–P25.

The cell lines were grown and maintained in Dulbecco’s modified Eagle’s medium (DMEM), supplemented with 10% fetal bovine serum (FBS), penicillin and streptomycin (10 u/mL), insulin (10 µg/mL) and fungizone (250 ng/mL), in a standard humidified incubator, at 37 °C in a 5% CO_2_ atmosphere. 

### 2.5. Gene Expression Analysis

Total RNA was extracted from TPC1, FTC133 and BCPAP thyroid cancer cells lines, using TRIzol reagent (Invitrogen, Waltham, MA, USA) and purified using an RNeasy Mini Kit (Qiagen, Venlo, The Netherlands). For gene expression, 500–1000 ng of total RNA was reverse transcribed using a High Capacity Reverse Transcription cDNA kit (cat no. 4374967; Applied Biosystems, Waltham, MA, USA), and the resulting cDNA was diluted and amplified with specific primers, according to the manufacturer’s instructions, using a real-time polymerase chain reaction (RT-PCR) system (Quant Studio 5; Applied Biosystems, Waltham, MA, USA). Gene expression levels were normalized using the *GAPDH* gene expression as an endogenous control. Results obtained were analyzed using the ΔΔCt method with SDS software (Applied Biosystems, Waltham, MA, USA). 

The taqman probes for detecting gene expression of human NOP53 (Hs00414236_m1, Catalog # 4448892) and internal control GAPDH (Hs02786624_g1, Catalog #4331182) were purchased from Thermo Fisher (Waltham, MA, USA). The amplicon length for *NOP53* was 118 bp and it spanned the exon boundary 1–2. The sequence of the probe is proprietary and would not be released.

### 2.6. Knockdown Studies

We conducted knockdown experiments using two different small interfering RNAs (siRNA) targeting NOP53 mRNA (cat# s26871 (si#1), cat# s26873 (si#2)) obtained from Ambion (Austin, TX, USA). Cells were reverse transfected in six wells with 50 nmol/L of siRNA, with the use of Lipofectamine RNAiMAX transfection reagent (Life Technologies, Frederick, MD, USA). All functional assays were carried out 48 h post transfection.

### 2.7. Site-Directed-Mutagenesis and Generation of Stable Cell Lines

The wild type ORF expression clone for *NOP53* gene (NM_015710.4) and the mutant ORF expression clone for *NOP53* gene with p. Asp31His (GAC → CAC) mutation were purchased directly from GeneCopoeia (Rockville, MD, USA). Both the wild type and mutant constructs were cloned in a pReceiver-Lv120 vector, a proprietary of Genecopoeia, Inc. The negative empty control vector (without wild type or mutant sequence) for pReceiver-Lv120 was used as a control for the experiments.

We added 50 ng of plasmid (control, wild type and mutant) into competent E. Coli cells following the transformation heat shock protocol. After growing overnight in a 100 µg/mL ampicillin containing LB-agar plate, we picked one colony from each control, wild type, and mutant-transformed cells. We inoculated these colonies into a LB-broth with ampicillin at 37 °C for approximately 24 h, and then we proceeded to isolate the plasmids from the transformed bacteria through Qiagen plasmid protocol (Hilden, Germany).

For the generation of stable control, wild type, and p. Asp31His variant cell lines, TPC1, FTC133 and BCPAP cells were transfected with 2.5 μg of the previously obtained plasmid DNAs in six wells with Lipofectamine 2000 (Life Technologies, Frederick, MD, USA). After 48 h of transfection, cells were selected with 1 μg /mL of puromycin containing growth medium for two weeks. During the selection of stably transfected cells, fresh growth medium containing the puromycin antibiotic was replaced every two days. After two weeks of selection, the transfected cells were analyzed for overexpression of wild type NOP53/ D31H-NOP53 by real time-quantitative PCR (qPCR) and Western blot. 

### 2.8. Clonogenicity and Proliferation Studies

We performed clonogenicity and proliferation assays using (a) the transient knockdown of wild type *NOP53* 48 h after transfection, and (b) the stable wild type and p. Asp31His variant overexpression, in TPC1, FTC133 and BCPAP cell lines. For the proliferation assay, 48 h after transfection, 200 cells/well were plated in quadruplets in six 96-well plates, and incubated at 37 °C in the previously described medium. Starting the following day, and every 24 h, one plate was frozen at −80 °C (from day 1 until day 6). After one week, the proliferation assay was performed with the six plates, using the Cyquant Cell Proliferation Kit with the manufacturer’s protocol (Invitrogen, Waltham, MA, USA). Fluorescence was measured using a Spectramax i3X, and cell proliferation was expressed as the number of cells, or fold change in number of cells normalizing by seeding at day 1. For clonogenicity assays, 48 h after transfection, cells were split and seeded onto the 6 well plates in duplicates. Prior to seeding the cells, the 6 well plates were coated with a previously autoclaved 0.1% gelatin in phosphate buffered saline (PBS). After 30 min, gelatin solution was aspirated and the cells were then plated. For TPC1, FTC133 and BCPAP, 600 cells were seeded and incubated at 37 °C for 7–15 days. The cells were fixed with 4% Paraformaldehyde (PFA) for 20 min and then stained with 0.05% crystal violet for 30 min. Images of the cells were captured using microscopy.

### 2.9. Western Blot Analysis

RIPA buffer was used for lysis of cultured cells. The protein concentration in samples/lysates was measured using The Pierce BCA Protein Assay Kit (Thermo Fisher, Waltham, MA, USA). Total protein lysates (25 µg for TPC1 cell line; or 30 µg in the case of FTC133 and BCPAP cell lines) were subjected to sodium dodecyl sulphate-polyacrylamide gel electrophoresis (SDS-PAGE), transferred to nitrocellulose membranes, and immunostained overnight at 4 °C, using the antibody of interest. Anti-NOP53 (1:1.000, #73225) was purchased from Cell Signaling Technology (Danvers, MA, USA). Anti-human β-actin (1:1000, Santa Cruz Biotechnology, Dallas, TX, USA) was used as a loading control. Membranes were incubated with appropriate secondary horseradish peroxidase conjugated IgG (anti-rabbit 1:3.000, Cell Signaling Technology; or anti-mouse 1:3.000, Santa Cruz Biotechnology). To prevent non-specific background binding of the primary and/or secondary antibodies to the membrane, we used 5% Bovine serum albumin (BSA) in 10× Tris Buffered Saline (TBS) with 0.1% Tween-20 for 60 min, as a blocking solution. Proteins were detected using enhanced chemiluminescence (ECL, Pierce Biotechnology, Waltham, MA, USA). 

### 2.10. Tissue Samples 

We collected tissue samples from the four affected members of Kindred 2 (Figure 1), after informed consent. All diagnoses were evaluated and confirmed by an Anatomical Pathology Consultant in thyroid cancer.

### 2.11. Immunohistochemistry Studies

We collected tumor samples from the four affected family members in Kindred 2 (Figure 1), containing both tumoral tissue and histologically normal tissue adjacent to the thyroid cancer. Tumor tissue samples were formalin fixed, embedded in paraffin, and 5-micron thick sections, that included both tumor and adjacent normal thyroid tissue, were cut for immunostaining. Sections were deparaffinized using standard procedures and immunostaining was performed using Dako EnVision kits, according to the manufacturer’s protocol (Agilent Technologies, Santa Clara, CA, USA). Primary anti-NOP53 rabbit monoclonal antibody was used at 1:200 dilution and incubated overnight at 4 °C (ab131002, antigen: synthetic peptide- aa 380–429; (Abcam, Cambridge, UK). The entire slides were magnified 200× using a ScanScope XT digital slide scanner and viewed using ImageScope software (Aperio Technologies, Vista, CA, USA). Manual counting of staining intensities in tumor tissue relative to control—the adjacent histologically normal thyroid tissue—was performed using the image processing package ImageJ [20].

### 2.12. Statistical Analysis

Statistical analyses were performed using the GraphPad Prism 7.0 software (GraphPad Software, La Jolla, CA, USA). An unpaired student t-test was used for comparison between groups and analysis of variance for multiple group comparison. Values are shown as mean ± standard error of the mean (SEM) or mean ± standard deviation (SD). Asterisks denote the following significance levels: * *p* < 0.05, ** *p* < 0.01. 

## 3. Results

### 3.1. Whole Exome Sequencing and Identification of a Variant in NOP53

We performed WES using peripheral blood DNA from four affected individuals from a kindred, with five cases of nonsyndromic FNMTC (Kindred 1). We did not find mutations in: genes previously described in syndromic FNMTC (*APC*, *PTEN*, *WRN*, *DICER1* and *PRKAR1A)*, rearranged genes (*NTRK*, *PPARG*), DNA repair genes (*XRCC1*, *XRCC3*), genes involved in the development of the thyroid gland (*PAX8*, *JAG1*, *CDC42*, *GSTM1*, *GSTT1*, *SRGAP1*, *TERT*, *THRB*, *AKT1*, *SEC23B*, *ESR2*, *NKX2*, *TBL1X*), genes previously described in the literature in nonsyndromic FNMTC (*HABP2*, *TTF1*, *THADA*, *SEC23B*, *FOXE1*, *KLLN*, *MAP2K5*), oncogenes (*RET*, *MET*, *KIT*, *MERTK*), nor genes that have been found in TC with somatic mutations (*BRAF*, *NRAS*, *TP53*, *CDKN2A*, *ALK*, *ATF4*).

We executed stringent filtering criteria (Table 1) to identify SNVs and INDELs that segregated with all the affected members from Kindred 1, identifying 58 variants. Five out of the 58 were in genes described as tumor-suppressor genes or proto-oncogenes in the literature, or genes possibly involved in cancer pathways. These five variants, located in different genes, were selected for further analysis. After Sanger sequencing, only two variants were confirmed: p. Thr190Met in *SH3BP1*; and p. Asp31His in *NOP53* (also known as *GLTSCR2*). The three remaining predicted changes were located in repetitive sequences in exonic flanking regions, but they were not confirmed by Sanger. The two final variants were studied by Sanger sequencing in 44 additional families with FNMTC (37 with two affected members, and seven with three or more affected members), as well as in an independent control group of 100 subjects. 

The variant p. Thr190Met in *SH3BP1* is described in the non-Finnish European population with an allele frequency of 0.007. We did not find this variant in any other kindred, but it was present in 3% of our control group (100 anonymous samples from blood donation bank), so we discarded it due to the high frequency in our population (>2%). 

**Figure 2:**
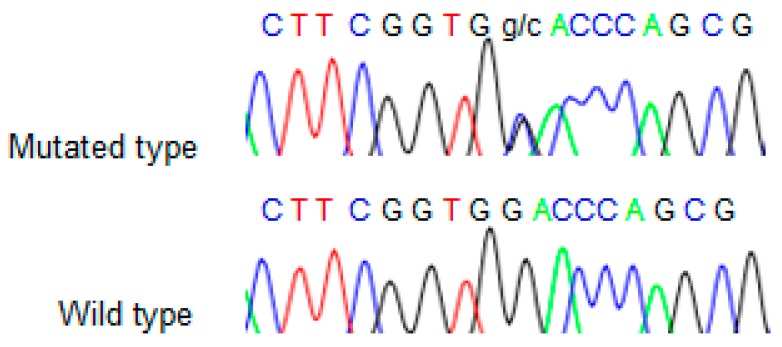
Sequence obtained from Sanger sequencing from one representative wild type and mutant sample.

**Figure 3:**
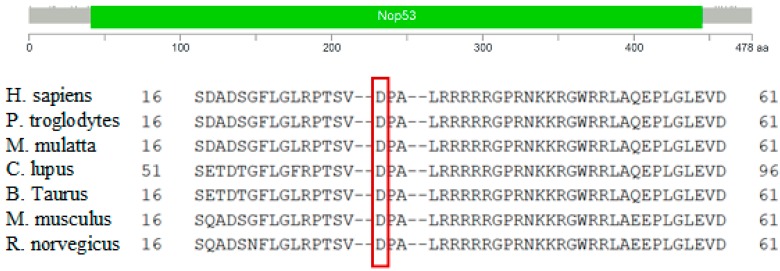
Protein domain architecture of NOP53 (GLTSCR2) and conservation of the p.Asp31 position across species. The red frame highlits the amino acid aspartic acid (D) at position 31.

We found the c.91G > C; p. Asp31His (D31H) variant (dbSNP: rs78530808) in *NOP53* gene (Figure 2). This variant was present in heterozygosis and segregated with all affected members in three families (Figure 1): in Kindred 1 (five out of five affected members), in Kindred 2 (four out of four), and in Kindred 3 (two out of two). This variant was not present in the non-affected (NA) spouse control II.1 (Kindred 1), nor in the NA spouse control II.3 (Kindred 2), who did not have any thyroid problem. This variant has been reported in ExAC, with an MAF of 1.8% in the European non-Finnish population. The Asp31 position is well-conserved across different species (Figure 3). 

We also checked the predicted effect of this variant in additional “in silico” algorithms (PolyPhen-2, mutation-taster), obtaining a probable damaging effect result in both (0.936 and 0.999 respectively). We also validated this variant by Sanger sequencing in our control population, and we found it with an allele frequency of 1.5% in our database (three out of 200 alleles presented the variant). None of the family members in Kindreds 1, 2 and 3 had a history of other primary cancers or clinical features suggestive of a syndromic FNMTC (Table 2). 

Finally, we decided to study the functional role of *NOP53* gene and the identified p. Asp31His variant. 

### 3.2. Knockdown of NOP53 Using siRNAs Reduces Cell Proliferation and Clonogenicity

**Figure 4:**
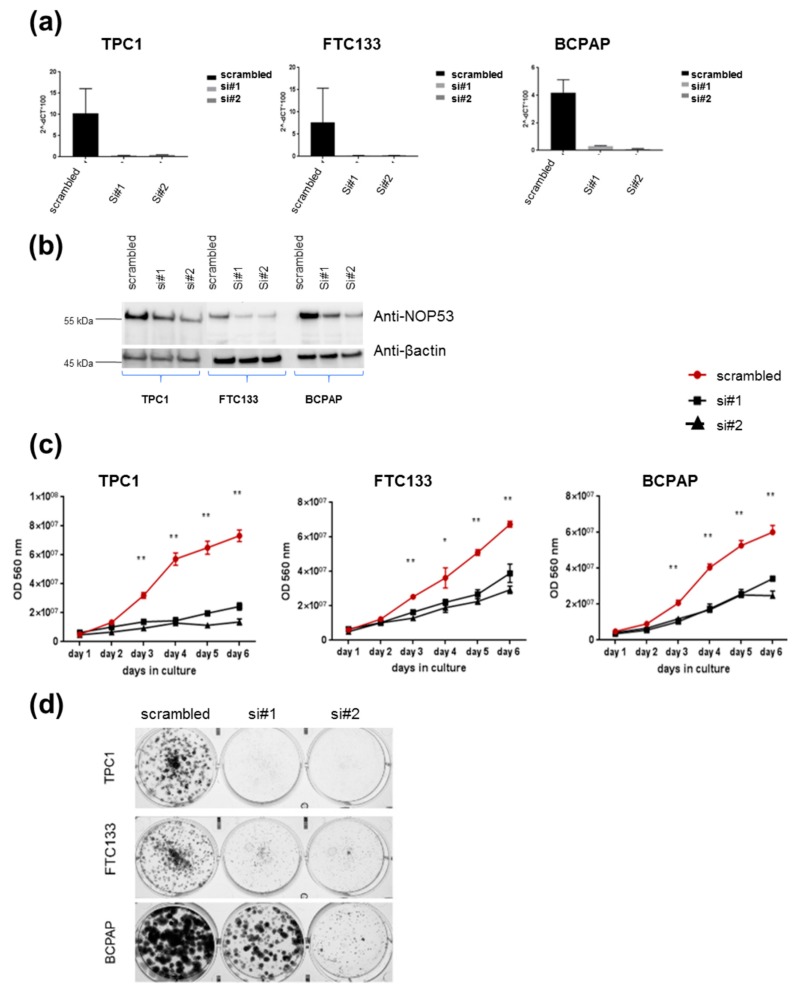
Knockdown of wild-type *NOP53* reduces cell proliferation and clonogenicity: (**a**) Validation of two different siRNAs (si#1 and si#2) targeting NOP53 gene expression in three different thyroid cancer cell lines (TPC1, FTC133 and BCPAP) using qPCR; (**b**) Validation of two different siRNAs (si#1 and si#2) targeting NOP53 protein expression in three different thyroid cancer cell lines (TPC1, FTC133 and BCPAP) using Western blots. The total protein lysates used were 25 µg for TPC1 cell line; and 30 µg for FTC133 and BCPAP cell lines. GAPDH and β-actin were used as an internal and loading control for qPCR and Western blot, respectively; (**c**) Transient knockdown of *NOP53* in three different cell lines with two siRNAs significantly reduced cell proliferation compared to negative control (scrambled), suggesting a proto-oncogenic function of *NOP53*; (**d**) Transient knockdown of *NOP53* in three different cell lines with two siRNAs significantly reduced cell clonogenicity compared to negative control (scrambled), suggesting a proto-oncogenic function of *NOP53*. * indicates adjusted *p* value < 0.05 compared to control. ** indicate adjusted *p* value < 0.01 compared to control. Error bars indicate standard deviation.

In order to understand the role of *NOP53* in TC cell lines, we performed functional studies upon transient knockdown experiments using siRNAs in these three TC cell lines. We validated the knockdown efficiency by qPCR and Western blot, both of which showed a significant decrease in NOP53 mRNA and protein expression (Figure 4, panels a and b, respectively). We also observed that knockdown of wild-type *NOP53* resulted in a significant reduction in cell proliferation and colony formation compared to the scrambled control. Accordingly, these results suggest that the suppression of *NOP53* reduces the growth in all the three tested cell lines (Figure 4, panels c, d). 

### 3.3. Overexpression of NOP53 Increases Cell Proliferation and Clonogenicity 

**Figure 5:**
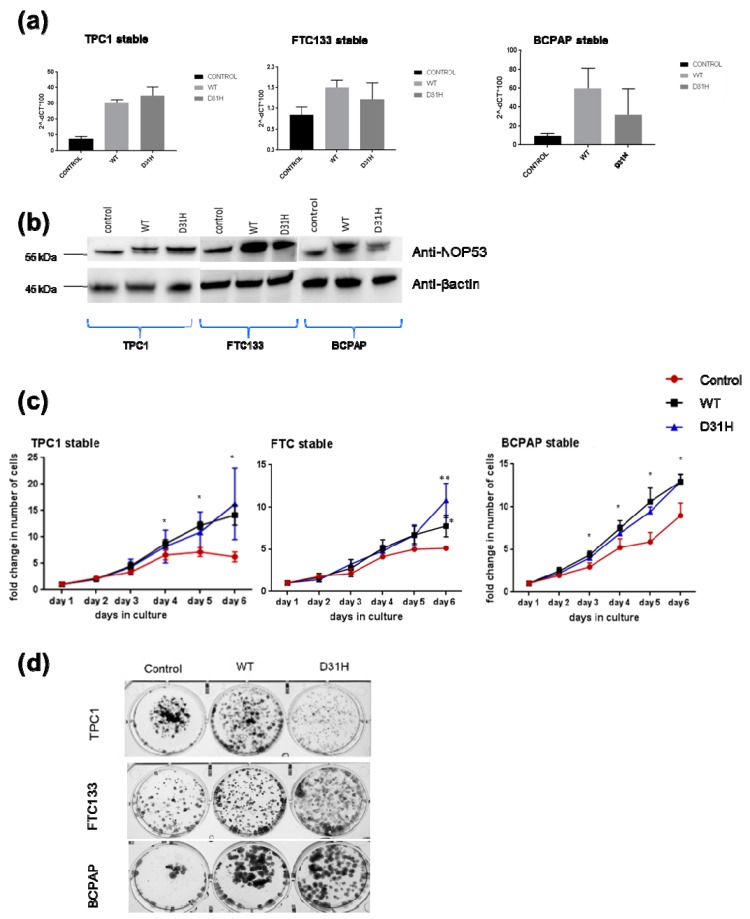
Effects of stable overexpression of *NOP53* in three cell lines (TPC1, FTC133, BCPAP): (**a**) Validation of stable overexpression of wild type (WT) and D31H mutant NOP53 in three cell lines by qPCR; (**b**) Validation by Western blot. The lower band corresponds to the endogenous protein expression, whereas the upper band represents the exogenous overexpressed protein. The total protein lysates used were 25 µg for TPC1 cell line, and 30 µg for FTC133 and BCPAP cell lines. GAPDH and β-actin were used as an internal and loading control for qPCR and Western blot, respectively; (**c**) Overexpression of WT and D31H mutant NOP53 significantly increased the cell proliferation in thyroid cancer cell lines compared to the vector control; (**d**) Overexpression of WT and D31H mutant NOP53 significantly increased the cell clonogenicity in thyroid cancer cell lines compared to the vector control. * indicates adjusted *p* value < 0.05 compared to control. ** indicate adjusted *p* value < 0.01 compared to control. Error bars indicate standard deviation.

We investigated the effect of overexpression through transfection of wild type *NOP53* gene and the p. Asp31His variant in the three cell lines. We confirmed the overexpression by qPCR and Western blot (Figure 5, panels a,b). Overexpression of *NOP53* significantly increased cell proliferation and colony formation in all three cell lines, compared to the empty-vector control, supporting a growth promoting role, which is consistent with the mentioned knockdown results (Figure 5, panels c,d). However, we did not detect significant differences in cell proliferation and clonogenicity between transfected *NOP53* wild-type and transfected p. Asp31His variant, except in FTC133 and BCPAP cells lines, where we observed more clones in transfected p. Asp31His cells.

### 3.4. NOP53 Immunohistochemistry in Tumor Samples

**Figure 6:**
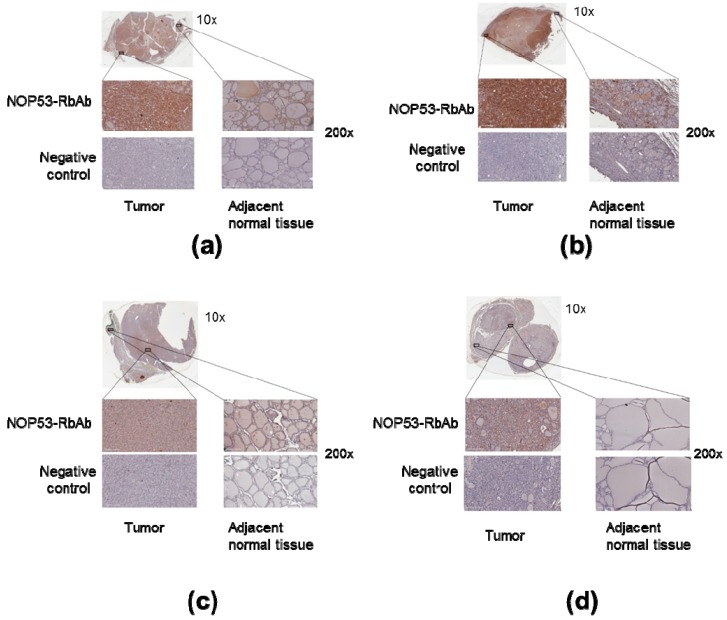
Overexpression of NOP53 in tumors from patients with FNMTC. Panels A through D show representative immunohistochemical staining for NOP53 in thyroid cancer samples from the four affected members of Kindred 2: (**a**) Corresponds to Patient III.1.; (**b**) Patient II.2.; (**c**) Patient III.2.; and (**d**) Patient II.1. Each panel contains an inlet (zoom 10×); two separate regions—from tumor tissue and adjacent normal thyroid tissue—of higher magnification images (zoom 200×); and two higher magnification images (zoom 200×) from a negative control specimen at a similar location. The top left represents tumor staining with NOP53-RbAb, the top right shows adjacent normal tissue staining with NOP53-RbAb, and the two bottom images are negative controls. We observed that the tumor tissue showed a higher expression of NOP53 compared to the adjacent normal thyroid tissue in the four patients studied.

We also analyzed NOP53 protein expression in tumor samples from the four affected family members in Kindred 2 using immunohistochemistry, as described previously. We noted that the tumor tissue from the samples showed more staining intensity and, therefore, higher expression of NOP53, compared to adjacent normal thyroid tissue—e.g., cells located amongst thyroid follicles—and negative controls (Figure 6, panels a–d). These findings suggest that NOP53 may be overexpressed in tumor tissue of patients with FNMTC.

## 4. Discussion

Several genes have been suggested to be implicated in non-syndromic FNMTC, however, until now, the results have not been conclusive or reproducible in additional families or studies [6,21,22]. We selected a kindred with five FNMTC-affected members (Kindred 1) to perform WES. We focused on variants with a predictable effect on the protein, that segregated with all affected members of the kindred, and was not present in spouse controls. We selected a population prevalence cut-off that was higher than the usual (≤ 2%), because the frequencies described in the general population do not always correlate with the real frequencies in our population, thus, we wanted to be more permissive with this filtering step, in order to not rule out possible interesting variants. We rigorously checked for each variant candidate, to find the real allele frequency in our control group. We also selected the variants that were present in genes described before in the literature as involved in cancer, because there were more chances they were implicated in thyroid cancer. Nevertheless, we cannot completely exclude the potential of the discarded variants, that were not studied further in the development of FNMTC. Establishing these filters makes the use of exome data biased, which would limit our study. 

We only found the candidate variant c.91G > C; p. Asp31His in *NOP53* gene to accomplish every filtering criteria, and it was present in all affected members of three different families with nonsyndromic FNMTC. 

*NOP53* gene is located in 19q13.33. It encodes a nucleolar protein which is involved in ribosome biogenesis [23]. It regulates the activation of p53/*TP53* in response to ribosome biogenesis perturbation, DNA damage and other stress conditions [24]. The participation of *NOP53* in the maintenance of nuclear morphology, chromosomal stability and mitotic integrity during nuclear division has been described, suggesting that *NOP53* expression may be a critical event in carcinogenesis [25]. It has been theorized that *NOP53* function depends on the cell type. Thus, several studies suggest that *NOP53* may have a tumor-suppressive function, since its expression was downregulated in renal cell carcinomas, and brain and breast cancer [26]. In contrast, high NOP53 expression was associated with poor prognosis in colon and esophageal cancers, suggestive of a growth-promoting or oncogenic function [27]. Interestingly, it has already been reported that *NOP53* (*GLTSCR2*) had higher expression in aggressive follicular carcinomas than in non-aggressive ones [28]. Among its main related pathways is PI3K/AKT signaling, and this pathway, together with the RAF/RAS/MEK/ERK signaling pathway, are the most commonly activated in thyroid cancer, leading to cancer initiation and/or progression [29]. 

*NOP53* gene is well conserved between different species, particularly the aspartic amino acid at position 31 (Figure 3), suggesting that variants in that position may have a deleterious effect. In addition, three “in silico” mutation algorithms—SIFT, PolyPhen-2, and mutation-taster—qualify this variant as probably damaging. This change is described in the non-Finish European population in ExAC and in The Genome Aggregation Database (gnomAD) v2.1 (non-cancer) with an allele frequency of 1.8%, and all the subjects in our study were Southern European. In our 100 person control group the variant appeared with an allele frequency of 1.5% (3/200). Hence, we have found an enrichment in families with FNMTC, compared to the general population or our control sample; it was observed in 6.7% of our families (3/45). Despite the high frequency of this variant in the general population, we have demonstrated that it co-segregates in all affected members of the three families, and it is not present in spouse controls. Furthermore, we calculated the likelihood of a random association between this mutation and thyroid affected patients, when considering the three families together, to be approximately 2 out of 1000 (1/512). 

In our knockdown experiments (Figure 4, panels c, d), we observed that, when we knock down *NOP53*, cell proliferation and clonogenicity clearly decrease, suggesting an oncogenic behaviour of *NOP53* in the three cell lines. In the overexpression experiments (Figure 5, panel c), we observed in the three cell lines that the wild-type and the mutant proliferate significantly more than the control, without significant differences between the wild-type and the mutant themselves. Thus, we hypothesize that, more than the specific p. Asp31His variant, *NOP53* may have an oncogenic role in thyroid cancer, and that this variant could be a mild-oncogenic mutation that has little surplus mitogenic effect when this gene is overexpressed. We should consider that not all the mutations present in a proto-oncogene are highly oncogenic. It is well known that the p. Val804M *RET* mutation, present in heterozygosis in the *RET* proto-oncogene, is rarely causative of medullary thyroid carcinoma, presenting high penetrance only when it is present in homozygosis [30]. A similar situation has been recently reported, implying a mild gain-of-function mutation in the *CaSR* gene [31]. If we assume that the p. Asp31His mutation is a mild gain-of-function mutation, it would only present its oncogenic potential if other genetic variants are present, representing a risk factor for the development of FNMTC.

Moreover, our immunochemistry studies (Figure 6) showed that NOP53 was overexpressed in tumor, as compared to adjacent normal tissue. In The Human Protein Atlas database [32], we found that, for NOP53 (GLTSCR2), the immunohistochemical staining in normal thyroid gland is mainly found in glandular cells, and with a medium antibody staining. By contrast, in our study, we observed that the tumor tissue from the affected samples showed higher expression of NOP53 (intense staining), compared to adjacent normal thyroid tissue. 

Altogether, our functional studies are consistent with an oncogenic function of *NOP53* in TPC1, FTC133 and BCPAP cell lines. We hypothesize that the *NOP53* gene could be involved in FNMTC, with an oncogenic role when presented in association with other genetic events in a polygenic scenario, and that p. Asp31His variant, and perhaps others in *NOP53*, may be mild mutations with no high penetrance, acting as a risk factor for development of TC. For example, the penetrance of *BRCA2* mutations in males is about 6% [33], therefore, it cannot be considered causative, but it multiplies the risk of developing the neoplasm. We have to remember that in most hereditary cancers, the number of high penetrance causative genes is usually only two or three—e.g., *BRCA1* and *BRCA2* in breast cancer, or *APC*, *MLH1* and *MSH2* in colon cancer—but many studies have tried unsuccessfully to discover a causative gene with a high penetrance for FNMTC. This could be because it is mainly a polygenic hereditary entity, and, in this context, it would be feasible that *NOP53* has an oncogenic role in some (but not all) families with FNMTC.

In conclusion, our findings suggest that *NOP53* could be a candidate modifier locus for FNMTC, although more studies are needed to deduce if the p. Asp31His variant or other variants are present in more families affected with FNMTC, as well as the pathways in which *NOP53* is involved in thyroid cancer. We think that our findings are interesting, as they can open research of the potential oncogenic role of *NOP53* in FNMTC for the first time, improving our understanding of the genetic mechanisms underlying this disease. However, we should consider that, as well as the *NOP53* gene, other genes are most likely involved in FNMTC.

## Figures and Tables

**Table 1:** SNVs and INDELs in Kindred 1 by filtering steps of whole-exome sequencing data.

Filter Criteria for Variants	Number of Variants (SNV and INDELs) after Filtering
Total number of variants	90,249
Present in all affected members of the kindred heterozygous; and coverage > 20	244
In exonic regions	118
Nonsynonymous (missense) or frameshift deletion/insertion	87
Deleterious SIFT score less than 0.05 or not available	82
SNVs/ INDELs ≤ 2% or not available in ExAC (European Non-Finish) and 1000 Genomes databases	58
Described as TSG or proto-oncogene	5
Confirmed by Sanger sequencing	2
Present in all affected members in at least another kindred	1

SNVs, Single nucleotide variants; INDELs, insertions and deletions; TSG, tumor suppressor gene.

**Table 2:** Clinical characteristics, pathological findings, and treatment in familial non-medullary thyroid cancer (FNMTC) affected members from Families 1, 2 and 3.

Kindred	K1	K1	K1	K1	K1	K2	K2	K2	K2	K3	K3
Patient	Patient II.2	Patient II.3	Patient II.4	Patient II.5	Patient III.1	Patient II.1	Patient II.2	Patient III.1	Patient III.2	Patient II.1	Patient II.2
Index Case	No	Yes	No	No	No	No	Yes	No	No	No	Yes
Age at Diagnosis	53	57	51	50	36	41	37	30	24	35	34
Presentation	Toxic MNG	MNG	MNG	Toxic MNG	Nodule on US screening	Thyroid nodule	Thyroid nodule	Thyroid nodule	Nodule on US screening	Thyroid nodule	Thyroid nodule
Histology	PTC	PTC	PTC	PTC	PTC	PTC	PTC	PTC	PTC	Hüthle cell carcinoma	PTC
Multicentricity	No	No	Yes	No	Yes	No	No	No	Yes	No	Yes
Bilateralism	No	No	Yes	No	Yes	No	No	No	No	No	No
Local Invasion	No	Yes	Yes	No	Yes	No	Yes	No	No	No	Yes
Stage ^1^ (TNM)	T1N0M0	T1N0M0	T2N0M0	T1NOMO	T1aN1bMO	T1N0M0	T2N0M0	T2N0M0	T2N0M0	T2N0M0	T1N1M0
Surgery	TT + LN dissection	TT + LN dissection	TT + LN dissection	TT + LN dissection	TT + LN dissection	TT + LN dissection	TT + LN dissection	TT + LN dissection	TT + LN dissection	TT + LN dissection	TT + LN dissection
Radioiodine Ablation (mCu)	Yes (311)	Yes (199)	Yes (118)	Yes (113)	Yes (28)	Yes (100)	Yes (300)	Yes (100)	Yes (100)	Yes (unknown)	Yes (unknown)
Radiotherapy	No	No	No	No	No	No	No	No	No	No	No
Disease Status ^2^	NED	NED	NED	NED	NED	NED	NED	NED	NED.	NED	NED

US, Ultrasound. PTC, Papillary-thyroid cancer. TT, Total thyroidectomy. LN, lymph node; NED, No Evidence of Disease. MNG, Multinodular Goiter. ^1^ Staging was based on the tumor–node–metastasis (TNM) classification of the American Joint Committee on Cancer 2016. ^2^ Disease status was assessed based on follow-up cervical ultrasonography, radioiodine scanning, and the stimulated serum thyroglobulin level.

## References

[1] Sahasrabudhe R., Stultz J., Williamson J., Lott P., Estrada A., Bohorquez M., Palles C., Polanco-Echeverry G., Jaeger E., Martin L. (2016). The HABP2 G534E variant is an unlikely cause of familial non-medullary thyroid cancer. J. Clin. Endocrinol. Metab..

[2] Moses W., Weng J., Kebebew E. (2011). Prevalence, clinicopathologic features, and somatic genetic mutation profile in familial versus sporadic nonmedullary thyroid cancer. Thyroid.

[3] Rowland K.J., Moley J.F. (2015). Hereditary thyroid cancer syndromes and genetic testing. J. Surg. Oncol..

[4] Kebebew E. (2008). Hereditary non-medullary thyroid cancer. World J. Surg..

[5] Vriens M.R., Suh I., Moses W., Kebebew E. (2009). Clinical features and genetic predisposition to hereditary non-medullary thyroid cancer. Thyroid.

[6] Hińcza K., Kowalik A., Kowalska A. (2019). Current knowledge of germline genetic risk factors for the development of non-medullary thyroid cancer. Genes.

[7] Wang X., Cheng W., Li J., Su A., Wei T., Liu F., Zhu J. (2015). Familial nonmedullary thyroid carcinoma is a more aggressive disease: A systematic review and meta-analysis. Eur. J. Endocrinol..

[8] Charkes N.D. (2006). On the prevalence of familial nonmedullary thyroid cancer in multiply affected kindreds. Thyroid.

[9] Malchoff C., Sarfarazi M., Tendler B., Forouhar F., Whalen G., Joshi V., Arnold A., Malchoff D. (2000). Papillary thyroid carcinoma associated with papillary renal neoplasia: genetic linkage analysis of a distinct heritable tumor syndrome. J. Clin. Endocrinol. Metab..

[10] Suh I., Filetti S., Vriens M., Guerrero M., Tumino S., Wong M., Shen W., Kebebew E., Duh Q., Clark O. (2009). Distinct loci on chromosome 1q21 and 6q22 predispose to familial nonmedullary thyroid cancer: A SNP array-based linkage analysis of 38 families. Surgery.

[11] Gudmundsson J., Sulem P., Gudbjartsson D., Jonasson J., Sigurdsson A., Bergthorsson J., He H., Blondal T., Geller F., Jakobsdottir M. (2009). Common variants on 9q22.33 and 14q13.3 predispose to thyroid cancer in European populations. Nat. Genet..

[12] Gara S.K., Jia L., Merino M., Agarwal S., Zhang L., Cam M., Patel D., Kebebew E. (2015). Germline HABP2 mutation causing familial nonmedullary thyroid cancer. N. Engl. J. Med..

[13] Ye F., Gao H., Xiao L., Zuo Z., Liu Y., Zhao Q., Chen H., Feng W., Fu B., Sun L. (2018). Whole exome and target sequencing identifies MAP2K5 as novel susceptibility gene for familial non-medullary thyroid carcinoma. Int. J. Cancer.

[14] Pereira J., da Silva J., Tomaz R., Pinto A., Bugalho M., Leite V., Cavaco B. (2015). Identification of a novel germline foxe1 variant in patients with familial non-medullary thyroid carcinoma (FNMTC). Endocrine.

[15] Weeks A.L., Wilson S.G., Ward L., Goldblatt J., Hui J., Walsh J.P. (2016). HABP2 germline variants are uncommon in familial nonmedullary thyroid cancer. BMC Med. Genet..

[16] Colombo C., Fugazzola L., Muzza M., Proverbio M., Cirello V. (2018). Letter regarding the article: “Multiple HABP2 variants in familial papillary thyroid carcinoma: Contribution of a group of “thyroid-checked” controls” by Kern et al. Eur. J. Med. Genet..

[17] Broad Institute GATK|Home. http://www.broadinstitute.org/gsa/wiki/index.php/The_Genome_Analysis_Toolkit,version3.4.0.

[18] ExAC Browser. http://exac.broadinstitute.org.

[19] 1000 Genomes|A Deep Catalog of Human Genetic Variation. http://www.internationalgenome.org/home.

[20] ImageJ. https://imagej.net/Welcome.

[21] Peiling S., Ngeow J. (2016). Familial non-medullary thyroid cancer: Unraveling the genetic maze. Endocr. Relat. Cancer..

[22] Valdes-Socin H., Palmeira L., Burlacu M.C., Daly A.F., Bours V., Beckers A. (2016). A familial non medullary thyroid carcinoma (FNMTC): Clinical and genetic update. Rev. Med. Liege.

[23] Sloan K.E., Bohnsack M.T., Watkins N.J. (2013). The 5S RNP couples p53 homeostasis to ribosome biogenesis and nucleolar stress. Cell Rep..

[24] Kim J.Y., Seok K.O., Kim Y.J., Bae W.K., Lee S., Park J.H. (2011). Involvement of GLTSCR2 in the DNA damage response. Am. J. Pathol..

[25] Lee S., Ahn Y.M., Kim J.Y., Cho Y.E., Park J.H. (2018). Downregulation of NOP53 ribosome biogenesis factor leads to abnormal nuclear division and chromosomal instability in human cervical cancer cells. Pathol. Oncol. Res..

[26] Moon A., Lim S.J., Jo Y.H., Lee S., Kim J.Y., Lee J., Park J.H. (2013). Downregulation of GLTSCR2 expression is correlated with breast cancer progression. Pathol. Res. Pract..

[27] Sasaki M., Kawahara K., Nishio M., Mimori K., Kogo R., Hamada K., Itoh B., Wang J., Komatsu Y., Yang Y. (2011). Regulation of the MDM2-P53 pathway and tumor growth by PICT1 via nucleolar RPL11. Nat. Med..

[28] Williams M.D., Zhang L., Elliott D.D., Perrier N.D., Lozano G., Clayman G.L., El-Naggar A.K. (2011). Differential gene expression profiling of aggressive and nonaggressive follicular carcinomas. Hum. Pathol..

[29] Kotian S., Zhang L., Boufraqech M., Gaskins K., Gara S., Quezado M., Nilubol N., Kebebew E. (2017). Dual inhibition of HDAC and tyrosine kinase signaling pathways with CUDC-907 inhibits thyroid cancer growth and metastases. Clin. Cancer Res..

[30] Loveday C., Josephs K., Chubb D., Gunning A., Izatt L., Tischkowitz M., Ellard S., Turnbull C. (2018). p.Val804Met, the most frequent pathogenic mutation in RET, confers a very low lifetime risk of medullary thyroid cancer. J. Clin. Endocrinol. Metab..

[31] Cavaco B., Canaff L., Nolin-Lapalme A., Vieira M., Silva T., Saramago A., Domingues R., Rutter M., Hudon J., Gleason J. (2018). Homozygous calcium-sensing receptor polymorphism R544Q presents as hypocalcemic hypoparathyroidism. J. Clin. Endocrinol. Metab..

[32] The Human Protein Atlas. 17/www.proteinatlas.org.

[33] Evans D.G., Susnerwala I., Dawson J., Woodward E., Maher E.R., Lalloo F. (2010). Risk of breast cancer in male BRCA2 carriers. J. Med. Genet..

